# Using fMRI non-local means denoising to uncover activation in sub-cortical structures at 1.5 T for guided HARDI tractography

**DOI:** 10.3389/fnhum.2014.00715

**Published:** 2014-09-11

**Authors:** Michaël Bernier, Maxime Chamberland, Jean-Christophe Houde, Maxime Descoteaux, Kevin Whittingstall

**Affiliations:** ^1^Department of Nuclear Medecine and Radiobiology, Faculty of Medicine and Health Science, University of SherbrookeSherbrooke, QC, Canada; ^2^Department of Diagnostic Radiology, Faculty of Medicine and Health Science, University of SherbrookeSherbrooke, QC, Canada; ^3^Computer Science Department, Faculty of Science, University of SherbrookeSherbrooke, QC, Canada

**Keywords:** fMRI, dMRI, non-local means, denoising, HARDI, tractography, seeding strategy

## Abstract

In recent years, there has been ever-increasing interest in combining functional magnetic resonance imaging (fMRI) and diffusion magnetic resonance imaging (dMRI) for better understanding the link between cortical activity and connectivity, respectively. However, it is challenging to detect and validate fMRI activity in key sub-cortical areas such as the thalamus, given that they are prone to susceptibility artifacts due to the partial volume effects (PVE) of surrounding tissues (GM/WM interface). This is especially true on relatively low-field clinical MR systems (e.g., 1.5 T). We propose to overcome this limitation by using a spatial denoising technique used in structural MRI and more recently in diffusion MRI called non-local means (NLM) denoising, which uses a patch-based approach to suppress the noise locally. To test this, we measured fMRI in 20 healthy subjects performing three block-based tasks : eyes-open closed (EOC) and left/right finger tapping (FTL, FTR). Overall, we found that NLM yielded more thalamic activity compared to traditional denoising methods. In order to validate our pipeline, we also investigated known structural connectivity going through the thalamus using HARDI tractography: the optic radiations, related to the EOC task, and the cortico-spinal tract (CST) for FTL and FTR. To do so, we reconstructed the tracts using functionally based thalamic and cortical ROIs to initiates seeds of tractography in a two-level coarse-to-fine fashion. We applied this method at the single subject level, which allowed us to see the structural connections underlying fMRI thalamic activity. In summary, we propose a new fMRI processing pipeline which uses a recent spatial denoising technique (NLM) to successfully detect sub-cortical activity which was validated using an advanced dMRI seeding strategy in single subjects at 1.5 T.

## 1. Introduction

Combining functional and diffusion magnetic resonance imaging (fMRI and dMRI, respectively) provides a unique non-invasive approach for investigating the structural architecture linking areas which are functionally active during cognitive processing. fMRI can be used to localize activation areas within cortex that represent changes in cortical blood flow, volume, and oxygen metabolism (Blood-Oxygenation-Level-Dependent or BOLD signal) associated with “active” brain tissue (Kwong et al., 1992; Turner, 1992; Bandettini et al., 1993; Menon and Kim, 1999; Buxton, 2002).

Even during relatively simple tasks, several activation areas can be seen, possibly reflecting networks of cerebral connectivity. To investigate this, diffusion MRI (dMRI), a non-invasive technique based on the observed anisotropic diffusion of water molecules along white-matter (WM) fibers, can be used to approximate and reconstruct WM tracts between such activation areas (Descoteaux et al., 2009; Descoteaux and Poupon, 2014). The probable direction of the diffusion in each voxel can be represented either by diffusion tensor imaging (DTI) (Basser et al., 1994; Basser and Pierpaoli, 1996; Pierpaoli et al., 1996) or more recently by high angular resolution diffusion imaging (HARDI) (Descoteaux et al., 2009; Tournier et al., 2012; Descoteaux and Poupon, 2014). HARDI allows for a more robust estimation of the fiber orientations in imaging voxels with complex and crossing fiber configurations, thus overcoming the limitations of DTI and allowing for more accurate tractography (Descoteaux et al., 2009; Tournier et al., 2012; Whittingstall et al., 2014). There is therefore a growing interest in combining dMRI and fMRI for studying large-scale networks *in vivo* (Zhu et al., 2013).

Sub-cortical areas of the brain such as the caudate, putamen, and thalamus are key areas involved in a wide range of cognitive tasks (Marzinzik et al., 2008; Saalmann and Kastner, 2011) and various neurological disorders (Grahn et al., 2008; Starr et al., 2011). Given their relatively deep locations, measuring their function is difficult with tools such as electro/magnetoencephalograpy (EEG, MEG). fMRI is potentially better suited for this, though, since these areas are prone to multiple MRI-related nuisances: susceptibility artifacts due to the interface of gray and white matter in these area (GM/WM interface), and partial volume effects (PVE) due to the mixture of GM and WM contained in the rather large fMRI voxels. Measuring reliable fMRI activity within them is challenging, particularly when acquired using a conventional 1.5 T scanner (Krasnow et al., 2003). Yet, given that these areas also carry important clinical implications, there is great interest in measuring their activity with a conventional low-field scanner, given that most clinical scanners are 1.5 Ts. Therefore, there is a need for an off-line analysis procedure that can help recover such activation.

One way to potentially uncover the relatively low amplitude BOLD signal in sub-cortical areas is to add a denoising step in the fMRI processing pipeline in order to amplify the signal-to-noise ratio (SNR). The most common and well known approach is to first spatially denoise fMRI data. As stated in Coupe et al. (2008), most denoising methods work by restoring the intensity value of each image voxel by averaging in some way the intensities of its neighboring voxels. Their major drawback is that they blur the structures of interest in the image (e.g., edges or small structures and textures). The most common technique to acheive this goal is the standard global Gaussian smoothing (Cox, 1996; Friston et al., 2000b; Smith et al., 2004; Strother, 2006). This approach replaces a voxel intensity value by a distance-weighted average of its surrounding neighbors such as a full-width half maximum (FWHM) of 5 mm, though this can vary across studies (Wink and Roerdink, 2004; Yue et al., 2010). Often, the FWHM is chosen independently of the data and set equal across the image. This can lead to problems, as the size and shape of activated regions may vary across the brain, leading to situations where certain regions are under-smoothed, while others are over-smoothed, potentially leading to false positives (Yue et al., 2010).

This has naturally led to data-dependent (edges preserving) denoising approaches, which rely on the principle that the restored value of a voxel should only depend on the voxels in its spatial neighborhood that belong to the same population or context, which is a method refered to as locally adaptive recovery paradigm (Coupe et al., 2008). One of these approach is non-local means denoising (NLM), which has recently shown powerful results in the image processing and computer vision literature (Buades et al., 2005). It is based on the idea that any natural image has redundancy, and that any voxel of the image has similar voxels that are not necessarily located in a spatial neighborhood. This new non-local recovery paradigm allows to combine the two most important attributes of a denoising algorithm: edge preservation and noise removal. NLM is based on this redundancy property of periodic, textured, or natural images to remove noise. In this approach, the weight involving voxels in the average, does not depend on their spatial proximity to the current voxel, like in a Gaussian smooth, but is rather based on the intensity similarity of their neighborhoods with the neighborhood of the voxel under study, as in patched-based approaches (Buades et al., 2005; Coupe et al., 2008; Wiest-Daessle et al., 2008; Manjon et al., 2010). Indeed, NLM has been shown to considerably improve MR image quality, resulting in enhanced voxel-based morphometry (VBM) and DTI- or HARDI-based tractograpy reconstructions (Coupe et al., 2008; Wiest-Daessle et al., 2008; Manjon et al., 2010). It is therefore possible that NLM may also improve fMRI activation detection in areas with low SNR and/or with complex data distributions. Moreover, since MR noise typically follows a Rician distribution (Wink and Roerdink, 2004), a Gaussian approach may not be optimal (Wink and Roerdink, 2006).

Other noise suppression approaches such as wavelet denoising or independent component analysis (ICA) attempt to separate signal and noise components of the BOLD signal (Smith et al., 2004; Wink and Roerdink, 2004). However, identifying noisy components is not trivial, often done manually, thus is subjective by being user-dependent. GLMdenoise is a technique that derive noise regressors from voxels to use in a general linear model (GLM) analysis of the data, but it is for denoising task-based fMRI data only (Kay et al., 2013). RETROICOR and CompCorr are also GLM-based techniques where nuisance components are learned and included as nuisance parameters within general linear models for BOLD and perfusion-based fMRI time-series data (Glover et al., 2000; Behzadi et al., 2007). These techniques perform well, but are dependant of the quality of the either manually selected or automatically acquired noise component. Additionally, these methods are focused on the BOLD temporal signal only and cannot be applied to spatial MRI acquisitions.

There is therefore interest in investigating alternative methods for denoising fMRI data in a less subjective manner without placing too many hard constraints on data distribution type or the type of acquisition. In the case of NLM, the standard deviation of the noise must be estimated in order to ajust a smoothing parameter in the original formula. The optimized NLM implementation (Coupe et al., 2008) can estimate automatically the noise standard deviation according to either a Gaussian or Rician distribution, allowing a fully automatic method with an adaptative smoothing strength.

One potentially useful application of NLM would be to improve fMRI activation detection in cortical and sub-cortical regions in order to reconstruct anatomical fibers linking them. In most cases, anatomical WM pathways are isolated by defining GM regions of interest derived from various structural atlases based on population averages such as the ICBM452 (Rex et al., 2003). fMRI-derived regions could build on this by identifying WM pathways based on subject-specific thalamic activity. One of these important pathways is the corticospinal tract (CST), which is comprised of fibers originating from the spinal cord and the cerebellum, passing through the pontine nuclei, up to the cerebellar peduncle, exchanging information to the thalamus and finally projecting to the motor cortex (Guye et al., 2003; Girard et al., 2012; Oguri et al., 2013). Lateral projections also connect to the motor strip as they cross the centrum semiovale (Fortin et al., 2012; Girard et al., 2012; Catani et al., 2013). It has been shown that CST tract size and density correlate with motor task performance (Philp et al., 2014), which is why finding subject-specific CST would be useful for studying rehabilitation in stroke, which often affects motor performance. Another well-known white matter pathway is the optic radiations, linking together the thalamus and the visual cortex near the calcarine sulcus (Benjamin et al., 2014).

To uncover these tracts, one optimal seeding strategy might be to use the fMRI activation site within the thalamus as a region of interest (ROI), which would act as a mid-seeding region between the cortical surface and the cerebellum for the CST, for example. It has also been suggested to use a two-ROIs approach for the reconstruction of fiber bundles in order to increase the percentage of valid trajectories (Huang et al., 2004). Other studies have attempted to launch initializations points (i.e., seeds) from a mid-plane generated from two pre-defined fMRI clusters (Morgan et al., 2009). In the context of an fMRI-based tract reconstruction, the common point of these studies is that uncovering activations in mid-point areas such as the thalamus greatly improves the quality of the recovered bundle. Also, the number of seeds used is often a trade-off between computation time and accuracy (Cheng et al., 2012). For example, whole-brain seeding is a computationally expensive process that requires time and can be memory consuming. In a neurosurgical context, intrusive brain tumors may displace or affect functional regions and hence we cannot rely on an anatomical brain function parcellation to choose seeding points. For this reason, launching seeds from a strategic point like fMRI-based ROIs is a potentially promising approach for precisely recovering desired fiber bundles without wasting computation time in irrelevant parts of the brain.

Overall, the contributions of this work are thus two-fold: (i) First, we show how a modified denoising pipeline allows the recovery of thalamic and putamen activations during a simple finger tapping paradigm and an eyes open closed task at 1.5 T. (ii) We also validate the uncovered activation clusters by looking at the underlying structural connections by means of an advanced seeding strategy.

## 2. Methods

### 2.1. Subjects

Twenty right-handed subjects (7 females, 13 right-handed, ages 18–30) were recruited for the study. Handedness was determined using the Edinburgh Handedness Inventory test (Oldfield, 1971). All subjects were native French speakers with no psychiatric or neurologic symptoms. The study was performed according to the guidelines of the Internal Review Board of the Centre Hospitalier Universitaire de Sherbrooke (CHUS).

### 2.2. Data acquisition

Imaging data were acquired using a 1.5 T SIEMENS Magnetom (Vision). Noise-reduction headphones and head cushions were used to minimize artifacts. Each session started with an anatomical T1-weighted 1 mm isotropic MPRAGE (TR/TE 1860/3.54 ms) acquisition, followed by a fMRI protocol and finally, with a dMRI acquisition. Details are given below.

#### 2.2.1. fMRI

We collected 3 separate fMRI datasets using a standard echo-planar imaging (EPI) sequence: 35 axial image slices, 64 × 64 matrix, TR/TE 2730/40 ms, voxel size 3.438 × 3.438 × 4.2 mm. Data were acquired in a box-car format, with subjects alternating between baseline and task conditions via short auditory cues. The three tasks used in this study were (1) a left (FTL) and (2) right (FTR) self-paced finger tapping sequence and (3) an eyes open-closed (EOC) sequence. For FTL and FTR, subjects alternated between epochs of 30 s rest, eyes closed, and 20 s of rapid tapping with thumbs and a predefined sequence of fingers: index, ring, middle, pinky. Subjects were asked to do the tapping sequence the fastest they could without making any mistakes. For the EOC acquisition, subjects alternated between periods of 30 s rest, eyes closed, and 20 s of simply keeping their eyes opened and fixating without blinking. The motivation for using the latter (as opposed to external visual stimulation such as a checkerboard) was to demonstrate how a relatively straightforward EOC task can yield robust thalamic activation maps with short acquisition times and without the need of external stimulation hardware/software. These points are very important in clinical settings.

#### 2.2.2. dMRI

Datasets were acquired using a single-shot echo-planar (EPI) spin echo sequence of 12 min (*TR/TE* = 11700/98 ms), with *b*-value of 1000 s/mm^2^ and 64 uniform directions. To reduce susceptibility distortions, GRAPPA parallel imaging was employed with an acceleration factor of 2. Other imaging parameters were matrix size of 128 × 128, 2 mm isotropic spatial resolution.

### 2.3. Data processing

#### 2.3.1. Functional data processing

**Figure 1:**
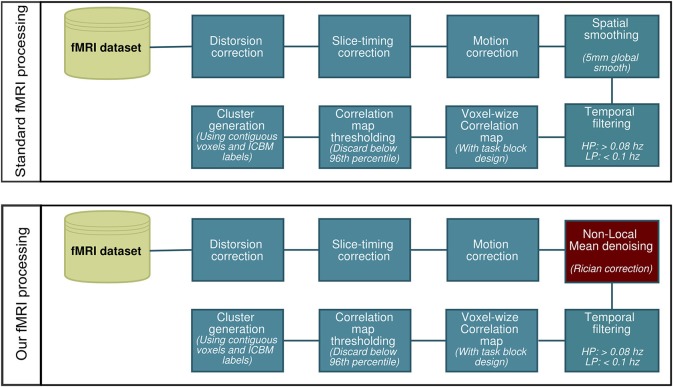
**Two slightly different fMRI processing pipelines for reconstructing fMRI activity**.

All fMRI analysis were carried out using AFNI (Cox, 1996). We first processed the data using a standard preprocessing pipeline (Cox, 1996; Friston et al., 2000b; Smith et al., 2004; Strother, 2006) consisting of slice timing and motion correction, 5 mm gaussian spatial smooth and band-pass temporal filtering (0.008–0.1 Hz). We then repeated the same process, though replacing the Gaussian spatial smooth with NLM denoising using Rician noise compensation (Descoteaux et al., 2008; Wiest-Daessle et al., 2008). This is illustrated in Figure 1. The activation maps were generated by computing the Pearson correlation coefficient between the hemodynamic response function (HRF) convolved stimulus box-car and the voxel time-series. These values were then converted to *z*-scores and thresholded at the 96th percentile (equivalent to a *z*-score of ~2.1). We then registered the activation maps to the T1-weighted images, which were then normalized onto the ICBM452 standard space (Rex et al., 2003) using ANTS (Avants et al., 2009). In each subject, the ROIs used for WM tractography were defined by labeling each thresholded voxel as “thalamic” or “cortical” via the ICBM atlas. The same was done for the group-averaged datasets.

#### 2.3.2. Local diffusion data processing

NLM denoising was performed on the raw diffusion data (Descoteaux et al., 2008). Diffusion tensor estimation and corresponding FA were estimated using MRtrix (Tournier et al., 2012). From this, the single fiber response function was estimated from all FA values above a threshold of 0.7. This single fiber response was used as input to spherical deconvolution (Tournier et al., 2007; Dell'Acqua et al., 2008; Descoteaux and Deriche, 2009; Tax et al., 2014) to compute the fiber orientation distribution function (fODF), also called fiber orientation distribution (FOD), at every voxel of the brain. In this work, we used the efficient implementation publicly available in MRtrix (Tournier et al., 2012) with a maximal spherical harmonics order of 8 and the default parameters. All dMRI derived metrics and models were upsampled to a 1 mm isotropic resolution. The T1-weighted image was then registered to the upsampled b = 0 image using ANTS. Quality control was done to make sure the registration was done robustly by manual inspection. A mask of the WM was derived from the T1-weighted image using FAST, from FSL (Zhang et al., 2001). To reconstruct the white matter fiber pathways, we used deterministic fiber tractography (*streamtrack* from MRtrix) on the field of fODF using multiple seeding and default tracking parameters (step size 0.2 mm, minimum/maximum streamline length 10/200 mm, minimum radius of curvature 1 mm, fODF amplitude cutoff at 0.1).

We performed NLM on all MRI datasets using the “Optimized blockwise non-local means filter for 3D MRI” toolbox developed by Pierrick Coupe and collaborators ^1^. A fast implementation of the NLM algorithm is publicly available in dipy.org (Garyfallidis et al., 2014). The goal is to remove the noise component of a voxel by exploiting the redundancy property of textured images: any voxel in the volume has similar voxels that are not necessarily located in a spatial neighborhood. In short, the technique attempts to match patches (defined as a reference voxel and its neighbors) with similar intensity spatial structure. Once matched, each patch is replaced by a weighted mean of all similar patches. In this approach, the weight involving each patch in the average does not depend on their spatial proximity but is rather define by the similarity of intensity patterns. NLM thus succeeds in both properties of a good denoising technique—edge-preservation and denoising quality—by automatically fine-tuning its parameters.

The NLM toolbox used in this study is based on overlapping blocks to define the voxel to optimize and their neighborhood in a way that is it computational efficient without affecting the quality of the final result. We used the default parameters [block size with a radius of a = 5 voxels, patch size with a overlapping radius of b = 1, automatic estimation of noise (standard deviation) with a Rician noise distribution]. The first two parameters were shown to be optimal and had an impact on computational time only. However, the automatic estimation of the noise could be replaced by a prior noise estimation or a manual input: the higher the standard deviation, the smoother the result will be, increasing noise suppression, but decreasing edge-preservation and increasing the chances of oversmoothing (Coupe et al., 2008). The 3D blockwise implementation of the NLM used in this work consists in (a) dividing the volume *V* into blocks with overlapping areas, (b) performing NLM restoration of these blocks, and (c) restoring the voxels values based on the restored values of the blocks they belong to Coupe et al. (2008).

A partition of the dataset volume *V* into overlapping blocks *B_k_* of size (2α + 1)^3^ is performed under the constraint that the blocks are overlapping. The non-overlapping sections of the blocks (patches *P_k_*) are of size (2β + 1)^3^. These blocks are centered on voxels *x_k_* which constitute a subset of the volume. For each block *B_k_*, a NLM restoration is performed as follows:

(1) $$NLM(B_{k})=\sum_{B_{i}\in V}w(B_{k},B_{i})\cdot B_{i},$$

with $\sum_{B_{i}\in V}w(B_{k},B_{i})=1$ and

(2) $$w(B_{k},B_{i})=\frac{1}{Z_{i}}\exp^{-\frac{\sum_{p\in P}||B_{i}{}_{p}-B_{k}{}_{p}|{|}^{2}}{h^{2}}},$$

where *h* is an exponential decay control parameter based on automatically learning the standard deviation and distribution of the noise (Gaussian or Rician), *Z_i_* is a normalization constant and *w*(*B_k_, *B*_i_*) defines the block *B_k_* likelihood to candidate *B_i_*. Implementation details are shown in Coupe et al. (2008).

#### 2.3.3. Seeding strategy

**Figure 2:**
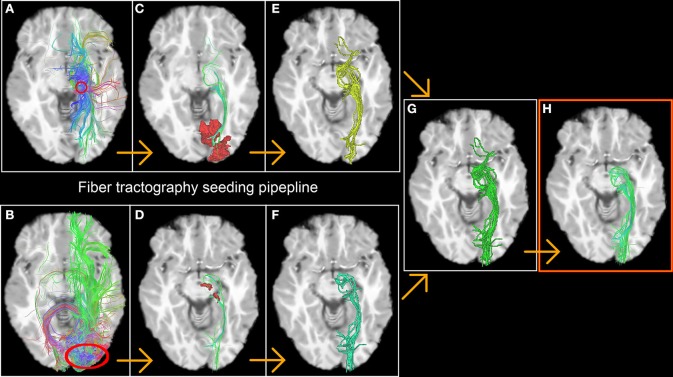
**The fiber tractography seeding process resulting in a functionnally derived reconstruction of the optic radiations**. The first step consists in generating streamlines by launching seeds from fMRI-derived ROIs in a bi-directional fashion [in this example, from the thalamus **(A)** down to V1 **(B)** and vice versa, red circles]. Only streamlines who reach the opposite fMRI ROI **(C,D)** were kept on to the next stage. A binary volume is then derived from both bundles **(E,F)**, and then united to create a new launching area to initiate the final seeds **(G)**. The resulting bundle is then filtered using the 2 fMRI ROIs previously described (red area **C,D**) to form the desired functionally-derived structural bundle **(H)**.

The first step consists of generating streamlines by launching seeds from fMRI-derived ROIs in a bi-directional fashion [for example, from the left thalamus (Figure 2A) down to left V1 (Figure 2B) and vice versa]. Only streamlines that reach the opposite fMRI ROI (Figures 2C,D) were kept for the next stage. From this, a binary volume *V* is derived from each set of streamlines (1 if a voxel is traversed by a streamline, 0 elsewhere) (Figures 2E,F) and both volumes are then combined to create a new seeding volume of interest *VOI* = *V*_1_ ∪ *V*_2_ used to initiate an additional seed region (Figure 2G). The resulting streamlines are then filtered using the 2 fMRI ROIs used in the previous steps (Figures 2C,D, red areas) to form the desired functionally-derived structural fiber bundle (Figure 2H). Note that for all of the above steps, we used a large number of randomly located seeds (9 per voxel) to ensure a better coverage of the fODF and representation of fiber bundles (Hagmann et al., 2008; Whittingstall et al., 2014).

From these bundles, we derived a set of binary maps that indicates the presence of a streamline in a voxel for each subject. These maps were normalized to the ICBM template using ANTS for group analysis. For each task, we finally computed the mean of all these binary maps to form a streamline occurrence score indicating the probability of the presence of a streamline for each voxel across the group of subjects: a strongly activated voxel meant that the probability of having a streamline in that voxel for a subject was high.

## 3. Results

### 3.1. fMRI tasks

**Figure 3:**
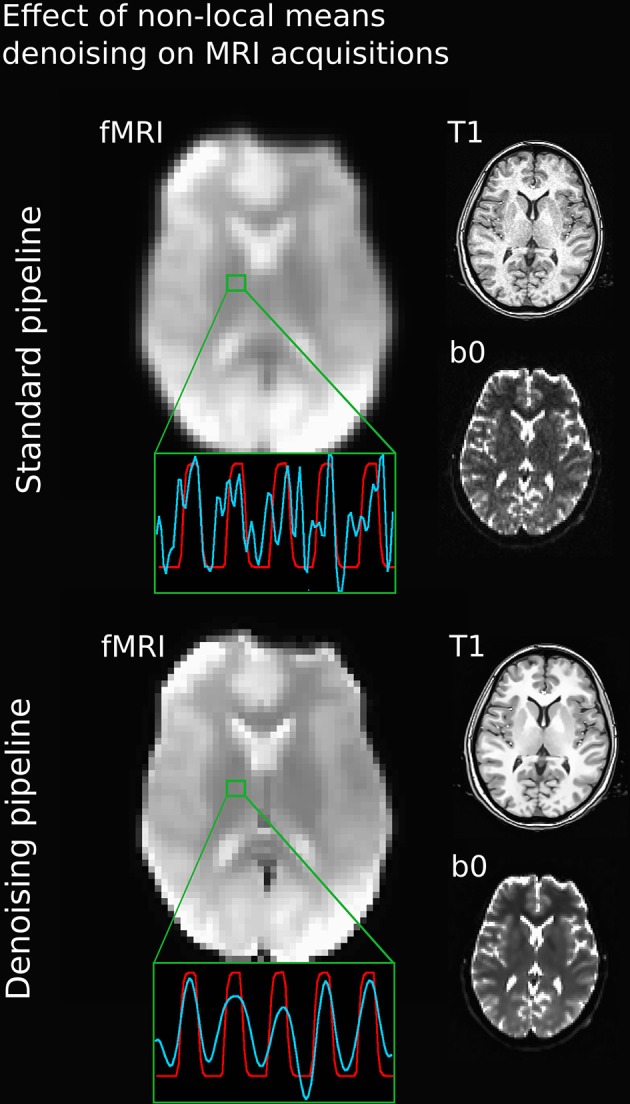
**Comparison between various MRI acquisitions with 5 mm Gaussian smoothed (top) and NLM-denoised (bottom) fMRI images**. Green square represents a single voxel within the thalamus with BOLD (blue) and stimulus (red) waveform after the processing pipeline. Note how all image types and BOLD signal appears sharper after NLM denoising. Even if a temporal filtering effect can be seen on the denoising pipeline, it is only the result of an effective spatial denoising: by removing some partial-volume effects, especially present in this area, the temporal signal appeared smoother, as rapid change of intensities (or spikes) had less chance of occurrence. The temporal filtering done afterward is then more efficient.

**Figure 4:**
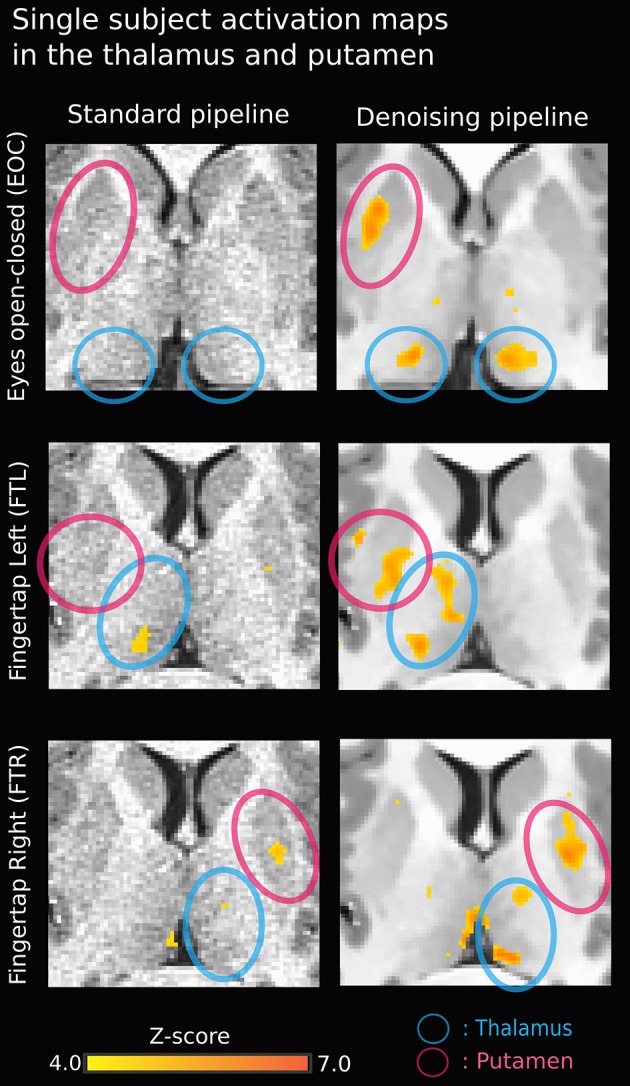
**Single-subject activation map using NLM denoising (right) yields stronger thalamic activation in all three tasks compared to standard approach (left)**. The activation in the bottom right image (FTR - denoised) near the left thalamus may be a veinous artifact.

**Figure 5:**
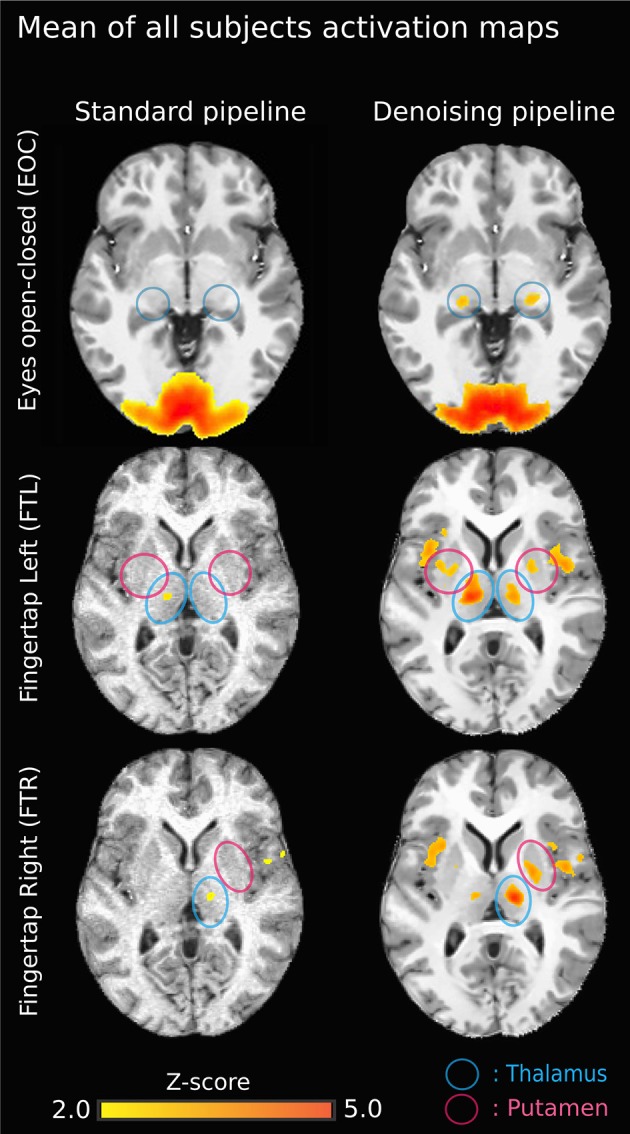
**Group-average activation maps (*z*-score normalized Pearson correlation) using the original pipeline (left) vs. NLM denoising pipeline (right) for each task**. Task-activation in sub-cortical areas is more robust using NLM. On the top left image (EOC - standard), the brain activation outside the cortex is mainly due to the gaussian smooth effect. Also, on the standard pipeline “EOC - NLM” image, there is a “spilled” effect caused by the Gaussian smooth, causing some activation to be outside of the cortex. Moreover, the thalamic activation (lateral geniculate nucleus, LGN) is increased (blue circles). Similar results are seen for both finger tapping tasks. The difference between FTl and FTR may be explained by the ratio between left and right-handed subjects. The now present activation in the external capsule may be the result of a veinous effect induced by denoising: NLM denoised the signal in veinous areas, therefore making it possible to isolate activations (with a strong percent change) in these structures.

**Figure 6:**
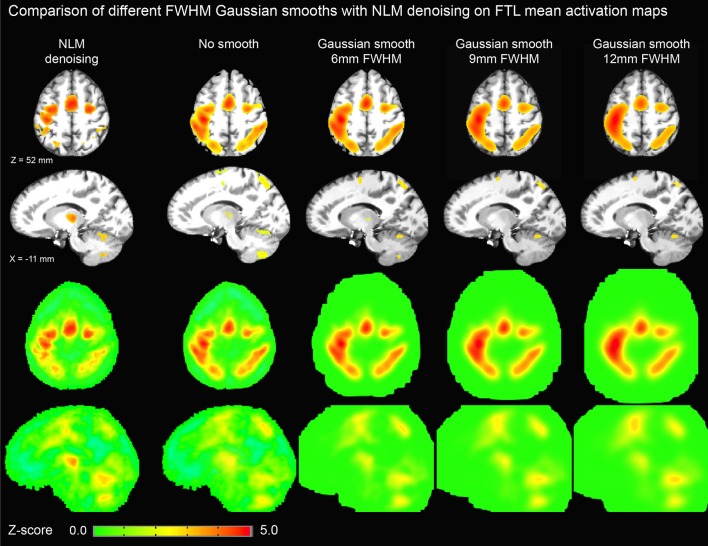
**Thresholded axial (top row) and sagittal (second row) representations of group-average activation maps using NLM and various degrees of Gaussian smoothing (no smoothing, 6, 9, and 12 mm FWHM)**. For comparison, unthresholded maps are shown in third and fourth rows. By increasing FWHM, cortical activation becomes more diffuse while thalamic activation is significantly reduced. On the other hand, NLM activation in the motor cortex is similar to that in the thalamus as evident in the unthresholded maps.

Figure 3 illustrates the effect of the NLM algorithm on our MRI datasets and on the BOLD activity in the thalamic area. For all fMRI tasks, NLM yielded significantly more activation voxels compared to the Gaussian pipeline using a standard 0.1 Hz low-pass filter (Students *t*-test, *p* = 0.0007 for pooled FTL and FTR, *p* = 0.0125 for EOC). An example of a thresholded (*p < 0.0001*, uncorrected) fMRI activation map is shown in Figure 4 for a single participant in all tasks (EOC, FTL, FTR), while group averages are shown in Figure 5. The main finding here is that the NLM denoising step greatly improves functional activation maps, particular in thalamic regions. We also compared the activation maps of NLM vs. Gaussian smooth using different FWHM (6 mm, 9 mm, 12 mm), which is shown in Figure 6. Overall, oversmoothing the datasets in the Gaussian pipeline could not uncover activation in the thalamus.

### 3.2. Functionally derived tracts

**Figure 7:**
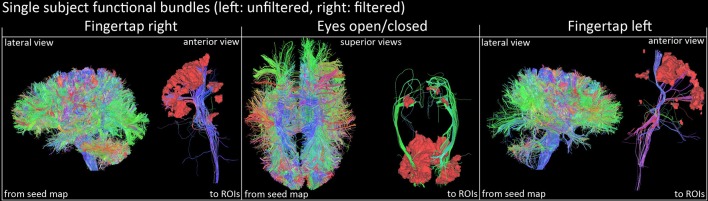
**Qualitative view of functional tracts derived from fMRI activation sites of a single subject (right images represent the filtered version)**. Streamlines were filtered according to their related functional task using basal ganglia activations and cortical ROIs (FTR/FTL: motor band, EOC: V1 area). Visualization was done using the FiberNavigator (Vaillancourt et al., 2011; Chamberland et al., 2014).

**Figure 8:**
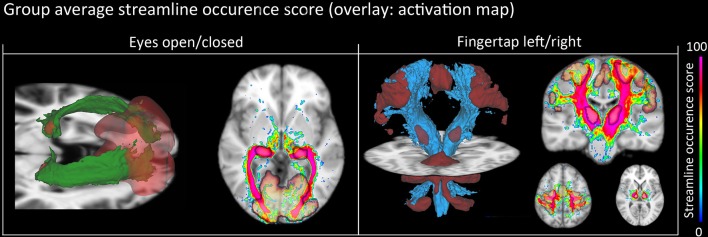
**Group-average WM pathway probability**. **Column 1:** 3D isovolumes (green: optic radiation, red: thalamic, and visual-cortex fMRI-derived activation sites). The axial slice shows the color-coded streamline occurrence score along with their respective fMRI activation maps. **Column 2:** 3D isovolumes (blue: CST, red: motor cortex, thalamus, and cerebellum) showing great correspondence with uncovered fMRI activation sites (motor strip, axial view). Visualization was done using the FiberNavigator (Vaillancourt et al., 2011; Chamberland et al., 2014).

Figure 7 shows a qualitative view of functionally-derived bundles for a single subject. Streamlines were obtained using the seeding strategy previously described in the **methods** section and in Figure 2. As one can notice, using the fMRI-activated clusters as ROIs allowed the recovery of the CST and OR both both FT and EOC tasks, respectively. Furthermore, on Figure 8, we show the streamline occurrence score (indicating the probability of having a streamline at each voxel across the group of subjects) along with the mean fMRI clusters from Figure 5. Tractography has not been conducted for the Gaussian denoising pipeline since thalamic activation was either too small (i.e., isolated single voxels) or not present at all.

## 4. Discussion

Most fMRI studies employ traditional Gaussian filtering which may suppress or attenuate activity in sub-cortical structures. Thus, we proposed an entirely novel approach for visualizing thalamic activity even on low-field scanner. Here, we showed that performing a NLM denoising technique allows the recovery of small sub-cortical activations, which were evaluated by a new HARDI tractography seeding strategy.

### 4.1. Effect of NLM denoising on fMRI data

NLM denoising was better suited at recovering thalamic activation sites compared to Gaussian spatial smoothing (Figures 4, 5). This effect was observed in both finger tapping and EOC, suggesting that our approach was able to recover activation in well-separated areas of the basal ganglia such as the caudate nucleus for the motor task and lateral geniculate nucleus (LGN) of the thalamus for the visual task (Figure 5). The locations of these activation sites were evaluated with our dMRI reconstructions, which showed the CST and optic radiation WM pathways that are known to link motor and visual parts of the thalamus (Figure 8).

One possible reason why NLM yielded better fMRI activation maps could be related to the global Gaussian smooth of the original pipeline: since the smoothing is applied equally without considering the amplitude of the BOLD signal, regions with low amplitude like the thalamus or with strong partial volumes effects may lead to further signal suppression. By removing this step and replacing it with a patch-based spatial method like NLM, we were able to successfully extract enough SNR to yield subject-specific and biologically-plausible activation maps. As far as we know, there is no important limitation or disadvantages to use NLM on fMRI datasets. However, as the technique is edge-preservating, if there is motion or residual artifacts in the dataset, NLM may enhance them instead of supressing them.

**Figure 9:**
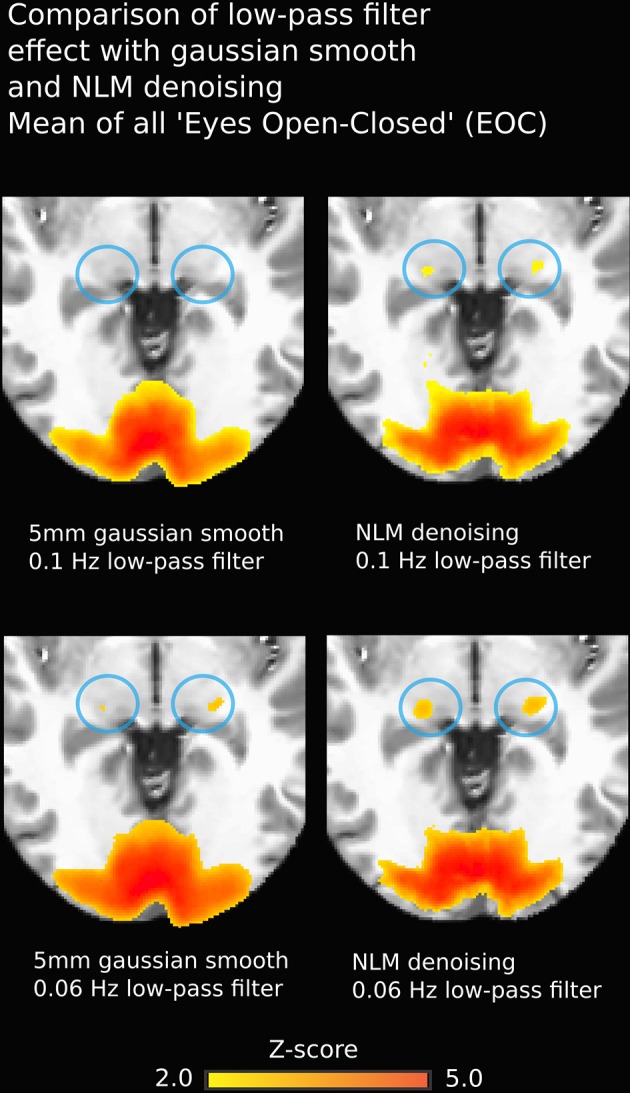
**Group-average fMRI activation during EOC**. Images on the left were obtained using a pipeline with Gaussian smoothing (FWHM = 5 mm) while images on the right are derived using the same pipeline where the Gaussian smoothing is replaced by NLM. Both approaches yield strong activation in bi-directional visual cortex, though thalamic reconstructions are more robust using NLM (blue circles). Effects are similar using 0.1 Hz **(top)** or 0.06 Hz **(bottom)** low-pass temporal filters.

Another non-negligible denoising aspect to investigate is temporal filtering, which uses intra-voxel signal redundancies to eliminate frequencies corresponding to noise (Friston et al., 2000a; Davey et al., 2013). The BOLD signal contains physiological noise such as synchronized cardiac and respiration signals (>0.1 Hz) (Krüger et al., 2001; Birn et al., 2006), which is why some studies, along with the usual high-pass filter to remove low-frequency trends, apply a low-pass filter with a threshold of 0.06–0.1 Hz (Niazy et al., 2011; Satterthwaite et al., 2013). However, low-pass filtering is debatable, as it may not be suitable for non-block design fMRI (Skudlarski et al., 1999; Strother, 2006). Regardless, we found that NLM outperformed Gaussian smoothing at different filter settings (see Figure 9). For example, in the EOC task (Figure 9), we can see that even without using a more aggressive temporal filter (0.1 Hz), we could still successfully extract activation in the thalamus. Group results for 0.06 Hz were the same as observed when using a 0.1 Hz low-pass filter (*p* = 0.0000005 for pooled FTL and FTR, *p* = 0.0043 for EOC). However, some activations could still be detected with the Gaussian, temporal filtered datasets. This illustrates how the Gaussian smooth is inappropriate in the standard approach, as the temporal filtering covers most denoising. This may also explain why the combination of the insufficiant spatial denoising by gaussian smooth and the low-pass temporal filter is innapropriate, while NLM reduces the chances of missing activations in key areas. With NLM, the spatial denoising could be sufficient, yet can be improved by a lower low-pass filter. Furthermore, NLM would greatly improve the signal processing of event-related fMRI processing, where temporal low-pass filtering cannot be applied as it may remove spikes of activation. As NLM was improved and adapted for dMRI using an angular q-space + spatial patch, NLM denoising for fMRI could be extended to better take into account the redundancy of the fMRI signal in the time domain. This will be investigated in future work.

### 4.2. Structural evaluation

fMRI activations in subcortical areas are often challenging to validate at 1.5 T, due to poor SNR. Using our group analysis of both the fMRI activation maps and streamline occurrence score (Figure 8), we qualitatively showed in an inter subject and inter modal analysis how the streamline occurrence score of the CST and optic radiations streamlines intersect with the fMRI ROIs (Figure 8). Our method thus allows the validation of functional clusters, since they are structurally related to the ongoing cognitive task. However, it cannot be directly used as a thalamus functional segmentation since the bundles obtained from our method were filtered by the fMRI activation sites, as mentioned on Figure 2.

Computation time is often the main bottleneck when performing dense whole-brain seeded fiber tractography when several millions of streamlines are wanted, especially from a clinical point of view (Chung et al., 2011). Also, one must be aware that fiber tracking algorithms are sensitive to initialization and therefore, the bundles generated by two different initializations may vary. Here, the seeding strategy employed can be viewed as a “localized” whole-brain fiber tractography (Figure 2). By placing seeds in an efficient manner (i.e., within the surroundings of the desired fiber bundle), we reduce unnecessary computations while maximizing coverage area. It has been showed in Buchanan et al. (2014) that performing seeding from the WM, rather than GM, has better test/retest performance. In our case, by initializing the seeds within a “bundle-shaped” ROI, any variability induced by seeding in GM-only (thalamus/cortical regions) was thus removed. By using the fMRI activation sites as a first initialization point, we were able to identify the exact fiber bundles passing through the thalamus that were responsible for the FTL, FTR, and EOC tasks.

## 5. Conclusion

We developed a modified denoising pipeline using non-local means denoising to recover thalamic and putamen functional activations during simple cognitive tasks at 1.5 T. It overcomes the well-known fMRI limitations for sub-cortical regions with low SNR. Moreover, the automated extraction of high quality fMRI-driven bundles was achieved by the means of an efficient seeding strategy. This method was easily reproducible on multiple subjects and tasks (Figure 8). We evaluated the thalamic fMRI activations using dMRI tractography with a group analysis of the pipeline reproducibility, recovering well-known motor and optic radiations tracts. This study sheds further light on the potential clinical implications where standard dMRI and fMRI analysis cannot be used and must be replaced by a simple and efficient way of labeling important cognitive regions and their tracts.

### Conflict of interest statement

The authors declare that the research was conducted in the absence of any commercial or financial relationships that could be construed as a potential conflict of interest.

## Footnotes

^1^https://sites.google.com/site/pierrickcoupe/softwares/denoising-for-medical-imaging/mri-denoising
